# Supplementary material for: MetaQTL: a package of new computational methods for the meta-analysis of QTL mapping experiments
Source: BMC Bioinformatics. 2007 Feb 8;8:49. doi: 10.1186/1471-2105-8-49 (PMC1808479; doi:10.1186/1471-2105-8-49)
Supplement: Additional File 7 — MetaQTL Package : jar file and tutorial. This Zip archive contains both the MetaQTL JAR file and the files of the tutorial. [file 1471-2105-8-49-S7.zip › org.inra.metaqtl/doc/index-files/index-5.html]

E-Index


|  |  |  |
| --- | --- | --- |
| **Overview** \| Package \| Class \| Use \| **Tree** \| **Deprecated** \| **Index** \| **Help** | |  |
| **PREV LETTER**   **NEXT LETTER** | **FRAMES**    **NO FRAMES**     **All Classes** |  |


A B C D E F G H I K L M N O P Q R S T U V W X Y Z 

---


## **E**

**E** - Variable in class org.metaqtl.util.OffspringManager.HaldaneWaddingtonProportion: **E\_coeff** - Static variable in class org.metaqtl.util.OffspringManager.HaldaneWaddingtonProportion: **edist** - Variable in class org.metaqtl.EMResult: The euclidean distance between the current and the previous vector of parameters (mu + pi) **EM\_CONTINUE** - Static variable in class org.metaqtl.algo.EMAlgorithm: The EM convergence status : continue **EM\_ERR** - Static variable in class org.metaqtl.algo.EMAlgorithm: The EM convergence tolerance. **EM\_FAILURE** - Static variable in class org.metaqtl.algo.EMAlgorithm: The EM convergence status : failure **EM\_ITER\_MAX** - Static variable in class org.metaqtl.algo.EMAlgorithm: The EM maximum number of iterations. **EM\_MIN\_DISTANCE** - Static variable in class org.metaqtl.algo.EMAlgorithm: The minimal mahalanobis distance between components. **EM\_OK** - Static variable in class org.metaqtl.algo.EMAlgorithm: The EM convergence status : success **EM\_START** - Static variable in class org.metaqtl.algo.EMAlgorithm: The number of replicates for a run of the EM-algorithm. **EMAlgorithm** - Class in org.metaqtl.algo: This class defines methods to perform the EM-algorithm for univariate mixture of gaussians with known variances. **EMAlgorithm()** - Constructor for class org.metaqtl.algo.EMAlgorithm: **EMCriteria** - Class in org.metaqtl: **EMCriteria()** - Constructor for class org.metaqtl.EMCriteria: **EMPTY\_BIOALLELE\_ARRAY** - Static variable in interface org.thalia.bio.entity.IBioConstants: **EMPTY\_BIOENTITY\_ARRAY** - Static variable in interface org.thalia.bio.entity.IBioConstants: **EMPTY\_BIOLGROUP\_ARRAY** - Static variable in interface org.thalia.bio.entity.IBioConstants: **EMPTY\_BIOLOCUS\_ARRAY** - Static variable in interface org.thalia.bio.entity.IBioConstants: **emRate(EMResult, double[], double[])** - Static method in class org.metaqtl.algo.EMAlgorithm: Compute the euclidean distance between the new parameters and the old ones to obtain a simple approximation of the EM convergence rate. **EMResult** - Class in org.metaqtl: **EMResult()** - Constructor for class org.metaqtl.EMResult: **EMResult(int, int)** - Constructor for class org.metaqtl.EMResult: **EMResultFactory** - Class in org.metaqtl.factory: **EMResultFactory()** - Constructor for class org.metaqtl.factory.EMResultFactory: **entities** - Variable in class org.thalia.bio.entity.bean.BioEntityCollectionBean: **eStep(double[], double[], EMResult)** - Static method in class org.metaqtl.algo.EMAlgorithm: The Expectation step : it just consists in updating the Z matrix, i.e the cluster membership probabilities. **ESTIMATED** - Static variable in interface org.thalia.bio.util.ILGroupPosition: **EstimatedLocusPosition** - Class in org.thalia.bio.util: Class Description Here **EstimatedLocusPosition(double, double)** - Constructor for class org.thalia.bio.util.EstimatedLocusPosition: **extractLocus(IBioLocus[], int)** - Static method in class org.thalia.bio.util.BioLocusTypes: This returns extracts in the given array of loci the locus which type matches the given one.

---


|  |  |  |
| --- | --- | --- |
| **Overview** \| Package \| Class \| Use \| **Tree** \| **Deprecated** \| **Index** \| **Help** | |  |
| **PREV LETTER**   **NEXT LETTER** | **FRAMES**    **NO FRAMES**     **All Classes** |  |


A B C D E F G H I K L M N O P Q R S T U V W X Y Z 

---
